# Evaluation of a preclinical photon-counting CT prototype for pulmonary imaging

**DOI:** 10.1038/s41598-018-35888-1

**Published:** 2018-11-26

**Authors:** Felix K. Kopp, Heiner Daerr, Salim Si-Mohamed, Andreas P. Sauter, Sebastian Ehn, Alexander A. Fingerle, Bernhard Brendel, Franz Pfeiffer, Ewald Roessl, Ernst J. Rummeny, Daniela Pfeiffer, Roland Proksa, Philippe Douek, Peter B. Noël

**Affiliations:** 1 Department of diagnostic and interventional Radiology, Technische Universität München, Munich, Germany; 2 Philips GmbH Innovative Technologies, Research Laboratories, Hamburg, Germany; 3 Department of Interventional Radiology and Cardio-vascular and Thoracic Diagnostic Imaging, Louis Pradel University Hospital, Bron, France; 4 CREATIS, CNRS UMR 5220, INSERM U1206 INSA-Lyon, France; 5 Chair of Biomedical Physics, Department of Physics & Munich School of BioEngineering, Technische Universität München, 85748 Garching, Germany; 6 Department of Radiology, Perelman School of Medicine, University of Pennsylvania, Philadelphia, PA 19104 USA

## Abstract

The purpose of this study was to investigate a preclinical spectral photon-counting CT (SPCCT) prototype compared to conventional CT for pulmonary imaging. A custom-made lung phantom, including nodules of different sizes and shapes, was scanned with a preclinical SPCCT and a conventional CT in standard and high-resolution (HR-CT) mode. Volume estimation was evaluated by linear regression. Shape similarity was evaluated with the Dice similarity coefficient. Spatial resolution was investigated via MTF for each imaging system. *In-vivo* rabbit lung images from the SPCCT system were subjectively reviewed. Evaluating the volume estimation, linear regression showed best results for the SPCCT compared to CT and HR-CT with a root mean squared error of 21.3 mm^3^, 28.5 mm^3^ and 26.4 mm^3^ for SPCCT, CT and HR-CT, respectively. The Dice similarity coefficient was superior for SPCCT throughout nodule shapes and all nodule sizes (mean, SPCCT: 0.90; CT: 0.85; HR-CT: 0.85). 10% MTF improved from 10.1 LP/cm for HR-CT to 21.7 LP/cm for SPCCT. Visual investigation of small pulmonary structures was superior for SPCCT in the animal study. In conclusion, the SPCCT prototype has the potential to improve the assessment of lung structures due to higher resolution compared to conventional CT.

## Introduction

Over the last decades, high-resolution computed tomography (HR-CT) has demonstrated to be a valuable tool for detection of lung diseases and exploration of the lung^1–5^. While air is carried to the lungs, it passes several structures, including trachea, bronchi, and bronchioles, which have features and structures within – or currently below – the spatial resolution of HR-CT systems. When it comes to pathological changes in the lung, HR-CT has a significant role in the diagnostic evaluation and therapy design^6^. One example is the detection and classification of lung nodules. Lung cancer is one of the most common diseases worldwide^7^. Siegel *et al*. estimate that in 2018 25% of all cancer deaths in the United States of America will be caused by lung cancer^8^. For classification of lung nodules, apart from growth rate, the shape and surface of the nodule is a clinically accepted marker to distinguish between benign and cancerous nodules. In comparison, malignant nodules are more likely to present themselves with irregular shapes, rougher surfaces, and speckled patterns^9^. A superior spatial resolution could not only improve the classification of small pulmonary nodules (≥4 mm) during the clinical routine^10^ but also improve the performance of software-based classification systems^11^. A different example is the early diagnosis of chronic obstructive pulmonary disease (COPD), which is gaining in importance worldwide^12^. In COPD airflow obstruction and airway inflammation frequently lead to a destruction of alveolar architecture with enlargement of distal airspaces. For early detection, HR-CT allows the clinician to assess wall thickness, which is currently only possible for larger airways^13,14^. Next generation HR-CT systems would allow a more robust evaluation of the larger and small airways. Thus, an earlier detection of COPD could become feasible.

Present clinical computed tomography (CT) systems are equipped with energy-integrating detectors with detector pixel dimensions in the range of approximately 1.0 mm. Recently, an ultra-high resolution CT – based on present detector technology – with pixel dimensions of 0.25 mm has been introduced with a focus on pulmonary and cardiovascular applications^15–17^. A different detection concept, which is currently investigated for its diagnostic range, are photon-counting detectors (PCD)^18,19^. The essential advantage of a spectral photon-counting CT (SPCCT) system is that incoming x-ray photons are directly converted in electronic signals and spectrally binned by analyzing the pulse heights generated in a semiconductor detection layer^20^. Recent developments showed promising results in the areas of abdominal^21–25^, cardiovascular^25–30^, neurological^31–33^, and nanoparticle imaging^34^. In addition to those possibilities, SPCCT will offer an improved spatial resolution due to smaller detector pixel sizes compared to the current clinical standard. The influence of electronic noise is significantly reduced in the direct-converting PCDs and can be considered as eliminated for the energy levels of incoming x-ray photons^35^. Hence, the reduced pixel dimension in PCDs comes along with a lower radiation exposure compared to (a similar reduction of detector pixel size with) energy-integrating detectors.

In this study, we investigate the resolution capabilities of a preclinical SPCCT prototype compared to a conventional CT by evaluating size and shape of lung nodules in a phantom model, measuring the modulation transfer function (MTF) and demonstrating lung structure visualization in an *in-vivo* acquisition of a rabbit.

## Materials and Methods

### CT acquisition

Images were acquired with a commercial 3^rd^ generation 256-row clinical CT scanner (iCT, Philips Healthcare, Best, The Netherlands) and a preclinical SPCCT prototype scanner. The clinical CT scans were matched to a CTDIvol of 7 mGy. The CT was operated with 120 kVp, 107 mAs, and two different focal spot sizes: a small focal spot resulting in high-resolution CT (HR-CT) and a standard focal spot (CT). The SPCCT was operated with a step and shoot acquisition protocol with 120 kVp, 100 mAs and 1 s gantry rotation time. The x-ray exposures of the CT and the SPCCT were chosen to equalize the Air KERMA. Acquisition parameters are listed in Table 1. Images were reconstructed with standard filtered backprojection (FBP).

**Table 1:** Acquisition and reconstruction parameters.

	CT	HR-CT	SPCCT
Voltage	120 kVp	120 kVp	120 kVp
Current	246 mA	156 mA	100 mA
Helical pitch	0.758	0.585	—
Rotation time	0.33 s	0.4 s	1.0 s
X-ray exposure	107 mAs	107 mAs	100 mAs
Acquisition mode	Helical	Helical	Axial (step and shoot)
Focal spot mode	Standard	Small	Small
Focal spot size	1100 µm × 1200 µm	600 µm × 700 µm	600 µm × 700 µm
Physical detector pixel size	1408 µm × 1140 µm	1408 µm × 1140 µm	500 µm × 500 µm
Reconstruction kernel	Filter E	Filter YC	ramp filter
Reconstruction voxel size	130 µm × 130 µm × 625 µm	130 µm × 130 µm × 625 µm	130 µm × 130 µm × 250 µm

### Spectral photon counting CT

The preclinical SPCCT scanner (Philips Healthcare, Haifa, Israel) is based on a clinical CT system (Brilliance iCT, Philips Healthcare, Haifa, Israel) providing a conventional x-ray tube and standard beam filtration but with a limited in-plane field of view of 168 mm and a z-coverage of 2.5 mm at isocenter. The scanner is equipped with hybrid multi-bin photon counting detectors, based on ChromAIX2 ASICs (application specific integrated circuit)^36^ combined with cadmium zinc telluride (CZT) as sensor material. The physical pitch of the detector pixels is 500 µm × 500 µm. The projected focal spot size is 600 µm in-plane and 700 µm in the z-direction.

### Lung phantom

**Figure 1:**
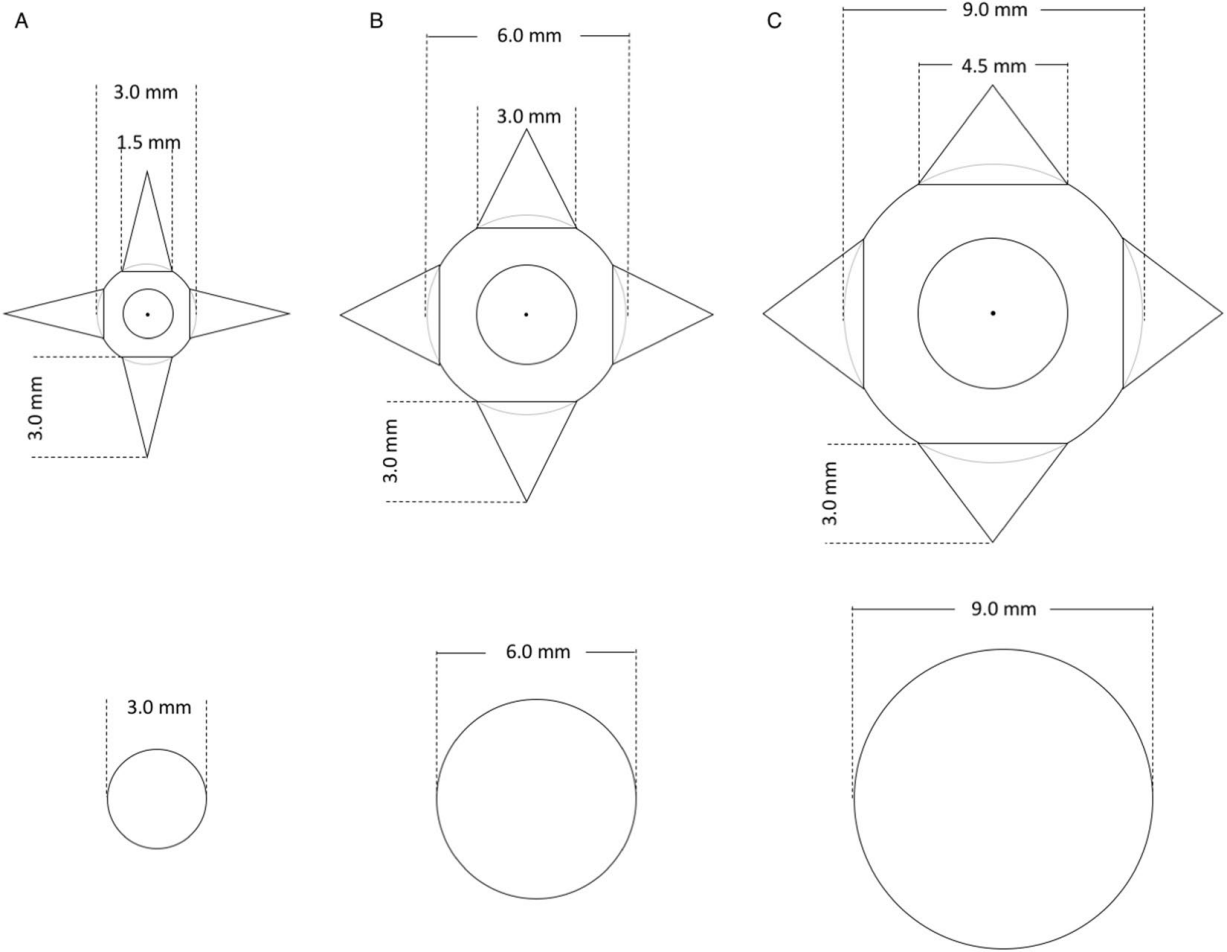
Description of the inserted nodules. First row: spheres with spikes; second row: spheres. Column (**A**) 3 mm nodules; (**B**) 6 mm nodules; (**C**) 9 mm nodules.

Patient data acquired with a conventional clinical CT system were used to build a digital model of a healthy lung. A threshold was applied to binarize the CT images and to differentiate the complex lung structure from the background. Lung nodules with two different geometries were simulated and inserted in the digital model–spheres to mimic benign nodules and spheres with spikes to mimic malignant nodules (Fig. 1), similar to the FDA lung-phantom inserts^37^. The three different sphere sizes had a diameter of 3, 6 and 9 mm. A board-certified radiologist assisted in the design process of the nodules and determined the location in the model for a realistic representation. The customized lung phantom was fabricated using an additive manufacturing technique of selective laser sintering based on polyamide. Measured Hounsfield Units (HU) of the vessels and surrounding walls of the lung phantom ([−*130*,−*90*] HU) were similar to values measured in clinical CT images ([*−130*, +*50*] HU). Due to the manufacturing process, the lung phantom was filled with powdered polyamide resulting in elevated HUs (about −580 HU). The background in the lung of the patient data was about −875 HU.

### Nodule segmentation

Lung nodules were segmented from the reconstructed image data (CT, HR-CT, SPCCT) for the evaluation of their volume and shape. The segmentation was performed with an in-house developed tool based on a numerical computing environment (MATLAB version R2017b, MathWorks, Massachusetts, USA). In each reconstruction, the estimated center of mass of each nodule was selected. A spherical volume of interest (VOI) around the selected center of mass was extracted from the images, with diameters of *d* + *1*.*5* *mm* for spheres and *d* + *6*.*5* *mm* for spheres with spikes (with *d* being the nodule size). The MATLAB-internal function *kmeans*, which is an implementation of the k-means clustering algorithm, was used with two clusters to separate the nodule structure from the background. Then, any non-connected component to the center of mass was removed from the segmentation. For each nodule, the segmentation was repeatedly performed three times to reduce the impact of the chosen center of mass on the measurements. Reported results for volume and shape quantification are the average over the three repeated segmentations.

### Nodule volume quantification

The nodule volume was determined by multiplication of the voxel count in one segmentation with the corresponding voxel size. The standard of reference was the segmentation performed on the digital lung phantom. Due to the realistic placement of the nodules inside the lung, connected parts to the center of mass of the nodules were included within a certain VOI (see section *Nodule segmentation*). Nodule volumes were evaluated with linear regression analysis and by comparing the different modalities to the standard of reference in a Bland-Altman plot.

### Nodule shape quantification

Due to different positioning of the lung phantom during scanning the images are not registered to each other and also not to the reference image. Therefore, nodule segmentations were semi-automatically registered to the reference segmentation of the three-dimensional (3D) printing template. In a first step, each segmentation was upscaled with cubic interpolation to isotropic voxel sizes of 0.14 × 0.14 × 0.14 mm^3^. In a second step, an expert in medical image processing measured rotation angles of the segmentations with respect to the reference. The segmentations were rotated around the x-, y- and z-axes to be in the same orientation as the reference. In a final step, two-dimensional (2D) cross-correlation of the mid-slices of the segmentation was used to shift to the same position as the reference.

After registration, the Dice similarity coefficient was computed to determine how well each modality can represent the reference nodules. The Dice similarity coefficient is given by

1 $$dice(A,\,{B}_{m})=2\cdot \frac{|A\,{\cap }^{}\,{B}_{m}|}{|A|+|{B}_{m}|},$$

where *A* is the reference template, *B*_*m*_ is the segmentation for the different modalities *m*, ${\cap }^{}$ denotes the intersection of two sets and $|\,\cdot \,|$ is the cardinal of a set. This results in the ratio of how many voxels in ${B}_{m}$ are correctly segmented. The Kolmogorov-Smirnov test showed a standard normal distribution for the differences between Dice coefficients of the different modalities. Thus, Dice coefficients for each of the nodules were compared between the different modalities with a paired-sample t-test (two-tail, significance level: 0.05).

### Spatial resolution

To evaluate the in-plane resolution of the CT and HR-CT images, the vendor specific phantom (Philips iCT head system, Philips Healthcare, Haifa, Israel) with a tungsten wire diameter of 200 µm was scanned at 120 kVp in the conventional CT. For the evaluation of the in-plane resolution of the SPCCT prototype scanner a comparable self-made phantom with a wire thickness of 100 µm was applied. The phantoms were aligned so that the wire was parallel to the rotation axis of the system and close to the rotation center.

The resolution was evaluated quantitatively utilizing the MTF. A small region-of-interest (ROI) around the wire was reconstructed using the same reconstruction filters and processing as for the images of the lung phantom. The MTF was then determined similar to the approach by Yu *et al*.^38^ Several image slices were averaged to reduce noise. The background was calculated as the mean of the image region excluding the wire and subtracted from the image. The resulting image was averaged radially around the wire to calculate a one-dimensional profile. A Hankel transform was applied to the one-dimensional profile to obtain the MTF^39^. The MTF was corrected for the finite size of the wire as described by Nickoloff^40^. Finally, the MTF was normalized to achieve unity at zero frequency.

### *In-vivo* experiment

A clinical HR-CT scan, selected from the departments Picture Archiving and Communication system (PACS), was visually compared to an *in-vivo* SPCCT acquisition of a New Zealand white rabbit (weight: 3.7 kg). The visual appearance was assessed by one experienced radiologist (board-certified; 4 years of experience). The study was approved by the French Department of Education and Research under the reference number *APAFIS#1732-2015091411181645 V3*. All experiments were performed in accordance with relevant guidelines and regulations.

### Patient population

Institutional review board approval was obtained prior to this study. Written informed consent was waived by the institutional review board (Ethikkommision der medizinischen Fakultät, Technical University of Munich, Germany) as all patients were included retrospectively. All scans were performed exclusively for clinical use with clinical standard protocols.

## Results

**Figure 2:**
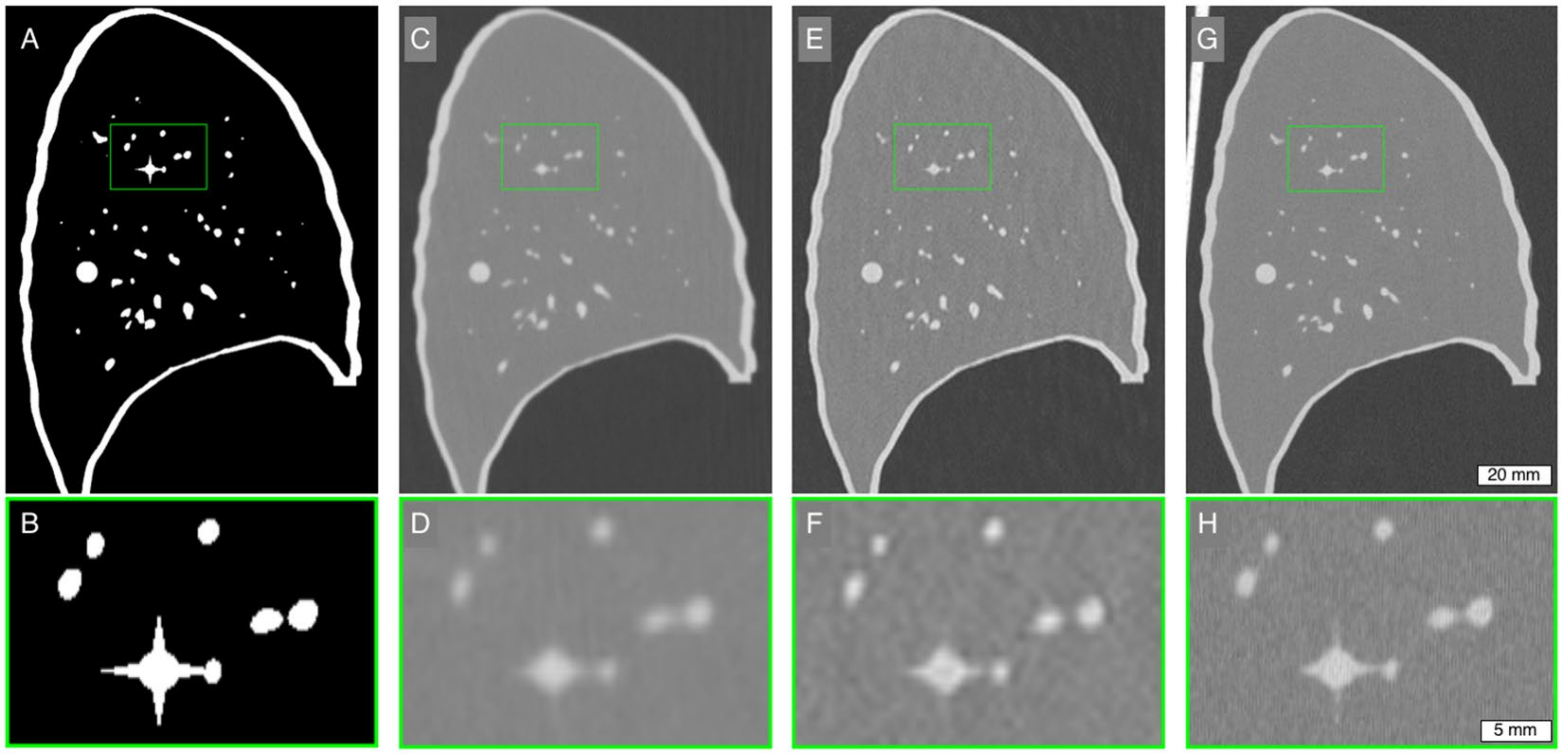
Comparison of different modalities with the reference. The upper row shows a sagittal slice through the lung phantom. The lower row is a magnification of the green rectangle in the corresponding image in the upper row. (**A**,**B**) template for 3D printing (reference); (**C**,**D**) CT; (**E**,**F**) HR-CT; (**G**,**H**) SPCCT. Note: There may be small variation in the structure of the different images due to the positioning of the phantom for each scan. Display window/level = 1700/−600 HU.

Figure 2 illustrates a sagittal slice of the lung phantom with a magnification of the area around the 3 mm sphere with spikes. Edges and boundaries were more prominent in images of the SPCCT compared to CT and HR-CT. Moreover, small details such as the spikes are closer in appearance to the reference.

**Figure 3:**
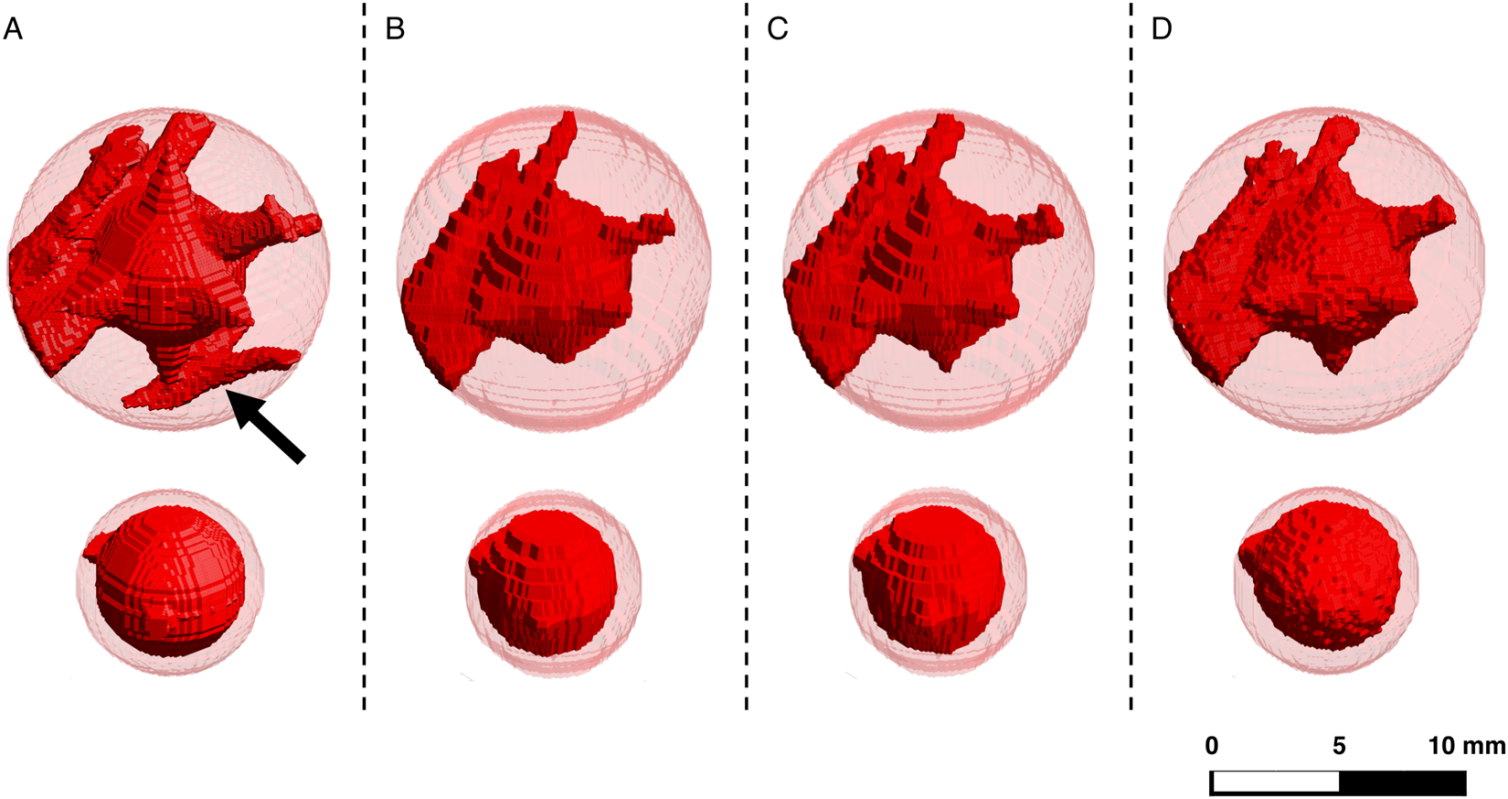
Three-dimensional volume rendering of the 6 mm nodule segmentations for the different modalities. The upper row displays the spheres with spikes, and the lower row shows the spherical nodules. Column (**A**) Reference used for 3D printing; (**B**) CT; (**C**) HR-CT; (**D**) SPCCT.

Figure 3 shows exemplary the segmentation for the 6 mm nodules. The spherical VOI is visualized in light transparent red and the segmented nodule is visualized in opaque red. Visually, one can observe that the 3D renderings from SPCCT data give the closest representation of the ground truth. However, a small blood vessel at the bottom of the sphere with spikes, indicated by a black arrow in the reference segmentation (Fig. 3A), is lost in every modality. The connection between the vessel and the peak of the bottom spike could not be identified in any modality. Hence, the vessel is not included in the segmentations of the different modalities.

**Figure 4:**
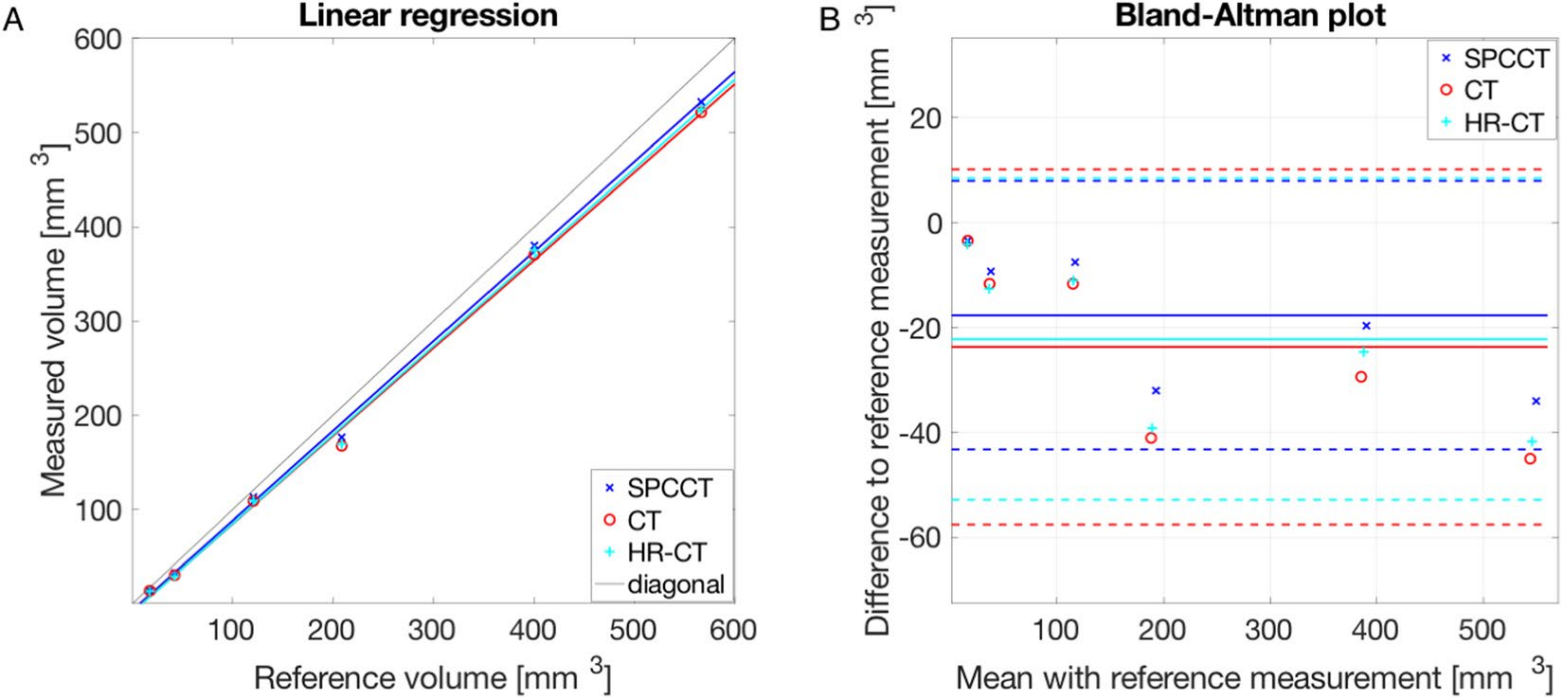
Linear regression and Bland-Altman plot of the volume estimation. (**A**) Linear regression, with the reference volume on the x-axes and the measured values on the y-axes. (**B**) Bland-Altman plot comparing the measured volumes to the reference volume. The plot shows a smaller mean error of SPCCT (blue solid line, −17.68 mm^3^) compared to CT (red solid line, −23.73 mm^3^) and HR-CT (cyan solid line, −22.23 mm^3^) with narrower boundaries (mean ± 1.96*SD; SPCCT: [−43.30; 7.94], CT: [−57.59; 10.14], HR-CT: [−52.91; 8.44]).

Volume estimation of the nodules showed an underestimation for all modalities. The linear regression gives the best results for SPCCT (slope: 0.952; intercept: −6.842 mm^3^) compared to HR-CT (slope: 0.942; intercept: −9.208 mm^3^) and CT (slope: 0.933; intercept: −8.622 mm^3^) with a root mean squared error (RMSE) of 21.3 mm^3^, 26.4 mm^3^, and 28.5 mm^3^ for SPCCT, HR-CT and CT, respectively (Table 2). Figure 4A shows a plot of the linear regression. The blue line for SPCCT is the closest to the diagonal line. Bland-Altman plots show a mean difference to the reference measurements of −17.68 mm^3^, −22.23 mm^3^ and −23.73 mm^3^ for SPCCT, HR-CT and CT, respectively. Ranges of differences are given by the 95% limits of agreement $[{\rm{\Delta }}-1.96\cdot \delta ,\,{\rm{\Delta }}+1.96\cdot \delta ]$, where Δ is the mean difference and $\delta$ is the standard deviation of the differences to the reference measurements. The ranges of differences were [−43.30; 7.94] mm^3^, [−52.91; 8.44] mm^3^ and [−57.59; 10.14] mm^3^ for SPCCT, HR-CT and CT, respectively.

**Table 2:** Summary of the linear regression.

	Slope (95% CI)	Intercept (95% CI) [mm^3^]	R-Square	RMSE [mm^3^]
CT	0.933 (0.873; 0.994)	−8.622 (−26.818; 9.574)	0.998	28.5
HR-CT	0.942 (0.882; 1.003)	−9.208 (−27.401; 8.984)	0.998	26.4
SPCCT	0.952 (0.901; 1.003)	−6.842 (−22.147; 8.463)	0.999	21.3

Linear regression was computed for the volume estimations over all nodule sizes and types. The values in the parentheses indicate the 95% confidence interval (CI).

Dice similarity coefficients were consistently superior for SPCCT (mean: 0.90) compared to HR-CT and CT (both, mean: 0.85), Table 3. The two-tail paired t-test showed a significant difference (P < 0.05) between the Dice coefficients of SPCCT and the values of HR-CT and CT (Table 3). The standard deviation of the Dice coefficients from three repeated segmentations indicate no substantial difference.

**Table 3:** Dice similarity coefficients for each nodule and modality compared to the reference nodules.

	Dice coefficient						Paired t-test (P-value)		
	9 mm sphere	9 mm star	6 mm sphere	6 mm star	3 mm sphere	3 mm star	CT	HR-CT	SPCCT
CT	0.920 ± 0.000	0.895 ± 0.001	0.907 ± 0.001	0.851 ± 0.000	0.799 ± 0.000	0.753 ± 0.006	/	0.962	0.002*
HR-CT	0.924 ± 0.000	0.901 ± 0.004	0.907 ± 0.002	0.861 ± 0.002	0.788 ± 0.001	0.745 ± 0.010	0.962	/	0.006*
SPCCT	0.970 ± 0.000	0.930 ± 0.003	0.935 ± 0.000	0.880 ± 0.002	0.870 ± 0.001	0.789 ± 0.005	0.002*	0.006*	/

Values close to one indicate a high similarity to the reference. Dice coefficients are given as mean of three repeated segmentations with standard deviation (mean ± SD). The paired t-test suggests a significant difference between the Dice coefficients for SPCCT and conventional CT (CT, HR-CT).

* Indicates a significant difference (P < 0.05).

**Figure 5:**
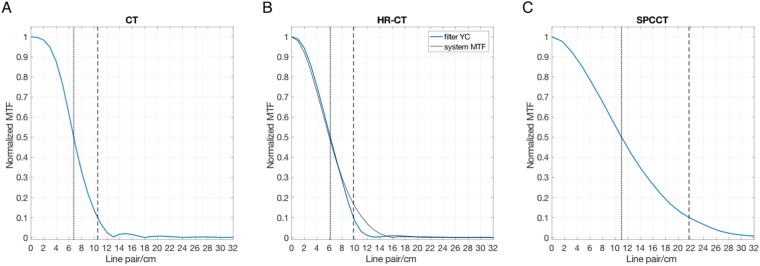
MTF of the different modalities. (**A**) Standard CT; (**B**) HR-CT; (**C**) SPCCT. The dotted line intersects the MTF at 50% and the dashed line intersects the MTF at 10%. The small oscillations in the MTF curve in (**A**) are caused due to unintended clipping of the data at −1024 HU. In (**B**) also the system MTF (with zero cutoff at 16 line pair/cm) for the HR mode was added (solid black line).

The MTF measurements are reported in Fig. 5. The 50% (10%) MTF cutoff was 6.7 (10.5), 6.1 (9.8) and 11.0 (21.7) LP/cm for CT, HR-CT and SPCCT, respectively.

**Figure 6:**
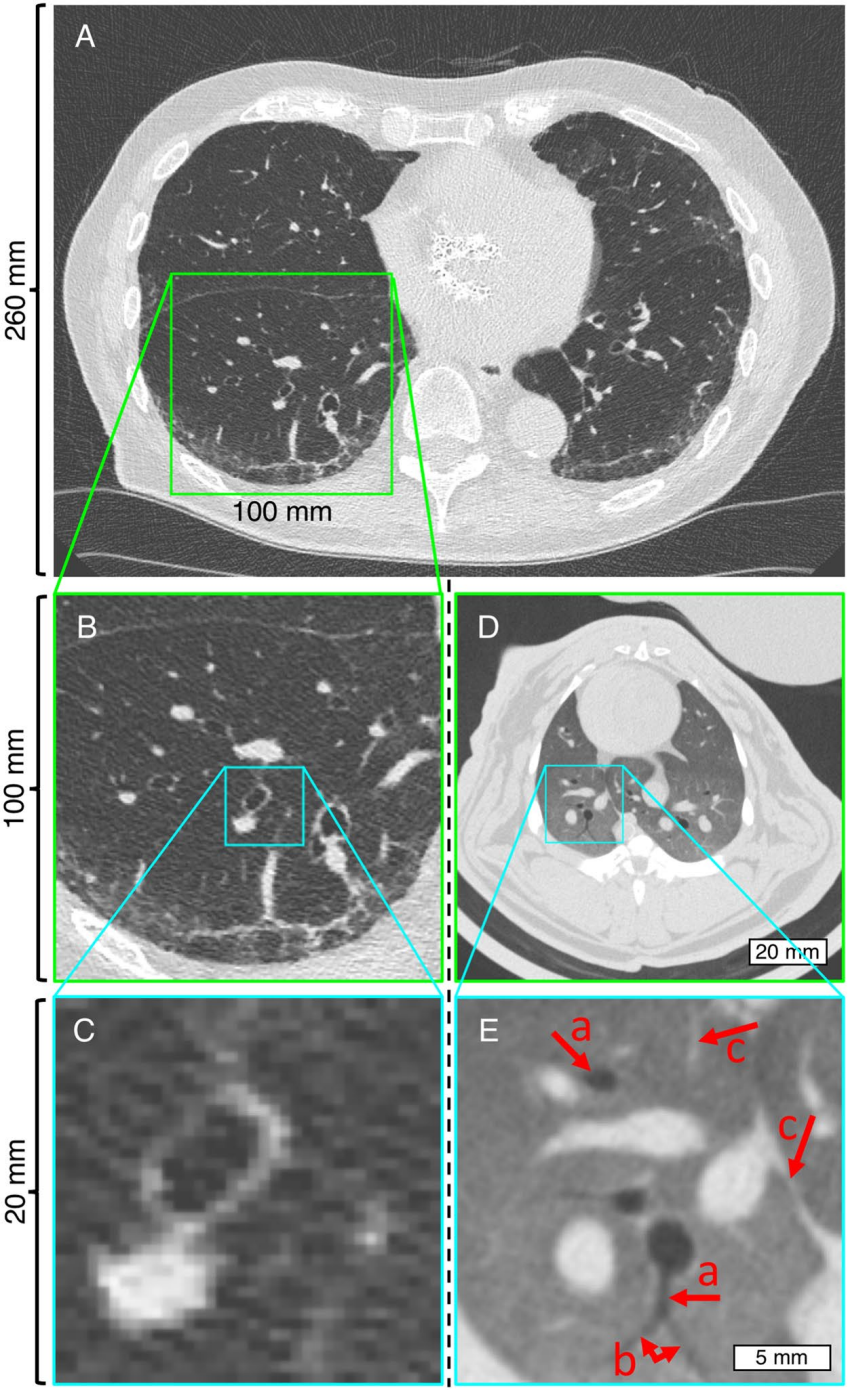
Comparison of images from HR-CT (**A–C**) and SPCCT (**D**,**E**). HR-CT shows a clinical CT scan of a human lung and SPCCT shows the lung of an *in-vivo* rabbit. Images with green and cyan frames have the same sizes, respectively. Image pixel were 0.56 × 0.56 mm^2^ for HR-CT and 0.13 × 0.13 mm^2^ for SPCCT. Display window/level = 1700/−600 HU.

Figure 6 shows a comparison between images acquired of an *in-vivo* rabbit with SPCCT and a patient acquired with HR-CT. With HR-CT, bronchi and bronchioles down to a diameter of 1.5–2 mm could be identified. An identification of smaller bronchioles (terminal, respiratory and lobular bronchioles) is not possible due to limited resolution. Some secondary pulmonary lobes and lobular arteries (1 mm in size) can be identified. With SPCCT, very small bronchioles with a diameter of below 1 mm (corresponding to a wall thickness of 0.15 mm) can be clearly identified (Fig. 6E, marked with arrow *a*). The branching of the dorsal bronchiole (Fig. 6E, arrow *b*) shows a typical separation of lobular bronchioles, suggesting that even lobular bronchioles can be visualized. Vessels to a diameter of below 0.4 mm can be identified (Fig. 6E, arrow *c*). Comparing images of HR-CT and SPCCT adjusted to the same size, vessels and walls of bronchioles are visualized more distinctively.

Overall, the subjective image quality of SPCCT-images is superior to HR-CT images regarding resolution and detectability of structures.

## Discussion

In this work, we investigated high-resolution imaging with a preclinical SPCCT prototype for pulmonary imaging in comparison to a commercially available CT system. We showed that the higher spatial resolution of SPCCT leads to a more precise assessment of lung nodules. Moreover, the visual investigation of small pulmonary structures was superior for SPCCT in the phantom and animal study.

Pourmorteza *et al*. illustrated that photon-counting detector CT (PCD-CT, a synonym for SPCCT) has the potential to provide high-resolution images with lower image noise compared to conventional CT^35^. On this note, various academic-industrial research collaborations are developing and evaluating multiple photon-counting detector concepts. While basic concepts and the ultimate goal between the different platforms are similar, individual parameters vary from concept to concept, e.g. detector pixel-size. As this is not the focus of this work, we would like to refer interested readers to the work of Willemink *et al*.^41^ In our study, we also observed superior high-resolution capabilities of SPCCT compared to conventional CT. However, we did not compare the noise levels of the different systems, because a fair comparison of the image noise would require the same spatial resolution for both systems. This would imply to reduce the spatial resolution of the SPCCT images, what is not intended in this study. It is known that higher spatial resolution results in more image noise given the same radiation dose. Reduced detector pixel sizes lead to a decreased number of photons reaching each detector element. The reduced statistics at the detector generates an uptake in noise. Moreover, in SPCCT high-energy photons and low-energy photons are weighted to contribute equally to the signal. In contrast, in conventional CT high-energy photons contribute relatively more to the signal than low-energy photons resulting in an uptake in image noise^41^. When it comes to low-dose CT another effect contributes to an increased noise level. The contribution of electronic detector noise increases in conventional CT. SPCCT, on the other hand, eliminates electronic detector noise to a certain extent by counting the photons resulting in lower image noise at same resolution.

There were several limitations of this work. We used FBP instead of advanced iterative reconstruction. Iterative reconstruction is known to deliver improved image quality compared to traditional FBP and could probably improve the results for both scanners, the clinical CT^42^ and the SPCCT^43^. However, due to regularization and other non-linearities, the evaluation of resolution becomes more challenging with iterative reconstruction^44^. With FBP, on the contrary, a more suitable comparison between conventional CT and SPCCT is feasible because effects of the reconstruction algorithms are reduced. Another limitation is the uncertainty in the production process of the lung phantom. Synthetic lung nodules were defined in the digital human lung model. For the 3D printing process, the digital lung model was used as input to the printer. During these processing steps, as well as during 3D printing, small errors might be propagated to the phantom due to interpolation or manufacturing processes. This might partly contribute to the discrepancy between the measured and the reference volumes. However, the RMSE in this work (21.3–28.5 mm^3^) is in the same range as reported by Zhou *et al*. in a similar study assessing lung nodules (21.6–28.3 mm^3^)^45^.

The presented results give a promising outlook to the high-resolution capabilities of the SPCCT prototype for pulmonary imaging. Higher spatial resolution, better assessment of lung nodule volume, and improved visibility of lung vessels compared to conventional CT and HR-CT were achieved. This would not only allow an earlier detection and more precise classifications of lung nodules but also improve the diagnostic confidence of radiologists assessing other pulmonary abnormalities, like COPD. In conclusion, the assessment of lung nodules could be improved with the presented preclinical SPCCT prototype. Especially the investigation of small pulmonary structures is improved due to higher resolution and the subjective higher image quality.

## Data Availability

The datasets generated during and/or analyzed during the current study are available from the corresponding author on reasonable request.
